# SARS-CoV-2 infection and exposure in cats and dogs in Romania

**DOI:** 10.3389/fvets.2025.1671681

**Published:** 2025-10-29

**Authors:** Andreea Paula Cozma, Mihaela Anca Dascalu, Ioana Buzdugan, Oana Tanase, Geta Pavel, Christelle Enond, Noëlline Guillou, Juliane Peronnet, Lucia Carmen Trinca, Smaranda Hristodorescu-Grigore, Anne-Geneviève Marcelin, Vincent Calvez, Stéphane Marot, Serban Morosan

**Affiliations:** ^1^Department of Exact Sciences, Faculty of Horticulture, “Ion Ionescu de la Brad” Iasi University of Life Sciences, Iași, Romania; ^2^Department of Public Health, Faculty of Veterinary Medicine, “Ion Ionescu de la Brad” Iasi University of Life Sciences, Iași, Romania; ^3^ROVETEMERG, “Ion Ionescu de la Brad” Iasi University of Life Sciences, Iași, Romania; ^4^Department of Preclinics, Faculty of Veterinary Medicine, “Ion Ionescu de la Brad” Iasi University of Life Sciences, Iași, Romania; ^5^UMS28, Sorbonne Université, INSERM, Paris, France; ^6^Sorbonne Université, INSERM, CNRS, Centre d'Immunologie et des Maladies Infectieuses (CIMI), Paris, France; ^7^Praxis Laboratory, Iași, Romania; ^8^Grigore T. Popa University of Medicine and Pharmacy, Iași, Romania; ^9^Institut Pierre Louis d'Épidémiologie et de Santé Publique (iPLESP), INSERM, Sorbonne Université, Assistance Publique-Hôpitaux de Paris (AP-HP), Hôpital Pitié-Salpêtrière, Service de Virologie, Paris, France

**Keywords:** SARS-CoV-2, COVID-19, pets, seroprevalence, Romania

## Abstract

SARS-CoV-2 has been described in more than 54 animal species, including wildlife, zoo animals and livestock. In the present study, conducted during 2021 and 2022 at the Faculty of Veterinary Medicine from Iasi, Romania, we studied the anthropogenic transmission of SARS-CoV-2 to pets by investigating active or prior infections of cats (*n* = 41) and dogs (*n* = 99) from the households of owners with confirmed COVID-19. Tests on an oropharyngeal swab from one cat revealed the presence of SARS-CoV-2 RNA 10 days after the onset of COVID-19 in its owner and another cat displayed SARS-CoV-2 seroconversion 15 days after the onset of COVID-19 in its owner but without the detection of SARS-CoV-2 viral RNA in its follow-up samples. Anti-N antibodies were detected in 7.2% (*n* = 7) of dogs and 12.5% (*n* = 5) of cats. All the seropositive cats were found to have SARS-CoV-2 neutralizing antibodies (NAbs) whereas only 42.9% (*n* = 3) of dogs displayed specific NAbs. These results are consistent with global reports, confirming the cross-species transmission of SARS-CoV-2. However, there is no evidence to suggest that companion animals are involved in the spread of SARS-CoV-2 to humans rather than simply being accidental hosts. Nevertheless, we describe several cases of potential anthropogenic infections during the pre-Omicron SARS-CoV-2 variant era.

## 1 Introduction

Animal coronaviruses display both digestive and respiratory tropism and are associated with a diverse range of diseases affecting the digestive tract or respiratory system, with systemic involvement in some cases (1). Feline coronaviruses (FCoV) belong to the *Alphacoronavirus* genus and have a digestive tropism, causing gastroenteritis in cats. However, a variant FCoV, feline infectious peritonitis virus (FIPV), has acquired mutations in the non-structural genes of the virus that have rendered it highly pathogenic through the acquisition of tropism for monocytes and macrophages. This change in tropism results in disseminated infection associated with viremia, cytokine storm, and peritonitis, with a fatal outcome in 95% of cases (2). There are two groups of canine coronaviruses. Group 1 consists of canine coronaviruses (CCoV) of genus *Alphacoronavirus* related to feline coronaviruses. These viruses cause enteritis, which can be severe in puppies, and a severe form resulting in generalized infection with deep organ involvement, known as pantropic coronavirus. Group 2 CCoV include the canine respiratory coronavirus, which belongs to genus *Betacoronavirus* and causes mild respiratory infections, such as rhinopharyngitis (1, 3). The aforementioned coronaviruses are specific to pets and do not cross the species barrier. However, a newly emerged human Coronavirus, named SARS-CoV-2, responsible for the 2019 pandemic has raised concern about the potential risk of transmission toward pets (4).

The first reported cases of coronavirus disease 2019 (COVID-19) in humans occurred in December 2019 in Wuhan, China. Severe acute respiratory syndrome coronavirus 2 (SARS-CoV-2) has since been shown to be a generalist virus with a wide host tropism (5) with a demonstrated ability to infect at least 54 non-human mammalian species, ranging from companion animals to wildlife (6, 7).

Consequently, concerns were raised at the beginning of the pandemic about the potential for SARS-CoV-2 transmission between companion animals and their owners and the potential risk of spillback from animal hosts to humans. Many of the observed animal infections resulted from contact with humans and did not lead to active transmission chains. Nevertheless, sustained transmission and spread at the level of animal populations were described for farmed mink and white-tailed deer (8). During replication within its host, SARS-CoV-2 acquires mutations throughout its genome, particularly in the region encoding the spike protein. Some of these acquired mutations may increase transmissibility enable the virus to evade neutralization by antibodies or increase its fitness in its new host (8). These concerns increased further with the emergence of more virulent strains of coronaviruses in dogs and cats, in the form of FIPV and the pantropic CCoV.

Several studies reported infections of pets with SARS-CoV-2 following exposure to infected humans in the USA, Hong Kong, Korea, Thailand, the UK, Belgium, Germany, Spain and France (9–19). However, most of these studies were performed during 2020, before the emergence of SARS-CoV-2 variants (20), and data for Eastern Europe remain limited.

In this study, we investigated cases of active or prior SARS-CoV-2 infection in companion animals belonging to owners who had COVID-19 during 2021 and 2022, when SARS-CoV-2 variants began to emerge and spread globally.

## 2 Method

### 2.1 Study population and specimen collection

A convenience sampling of dogs (*n* = 99) and cats (*n* = 41) seen over a 16-months period (from April 2021 to July 2022) at the Iasi Faculty of Veterinary Medicine belonging to owners with ongoing or prior confirmed COVID-19 (verified by SARS-CoV-2 RT-PCR or rapid immunochromatographic testing) was performed. Written consent to participate was obtained from the owners, regardless of the primary reason for presenting the animal at the clinic. The total number of samples was not balanced between the two species, as this aspect could not be controlled within the faculty clinics. Furthermore, due to refusal of an unexpected number of pet owners to participate in the study, a discrepancy in sample number is seen. For each animal included in the study, a questionnaire was completed, recording age and sex which were considered relevant variables. Additional information was also collected, including breed, vaccination and deworming status, lifestyle (indoor, outdoor, or mixed), presence of other animals or species in the household, travel history with the owner in the past year and general physical examination findings. Samples were collected with oropharyngeal (OPS), nasal (NS) and/or rectal (RS) swabs, transferred to individual virus transport media and stored at −80 °C for subsequent nucleic acid extraction. Serum samples were collected into venous blood collection tubes, centrifuged for 15 min at 1,500 rpm, aliquoted and stored at −80 °C until the performance of serological assays. Samples were collected by a trained veterinarian, in accordance with Romanian health regulations and the requirements of the Iasi Faculty of Veterinary Medicine for animal research. This study was approved by the Scientific Research Ethics Committee of the Iasi Faculty of Veterinary Medicine (Ref. 0001/2021).

### 2.2 Clinical examination and diagnostic tests

Diagnoses of canine parvovirus (CPV) and canine distemper virus (CDV) infections were confirmed/excluded by rapid quantitative immunochromatographic tests on V-Check equipment. Rectal swabs were collected and tested for the presence of antigens for CPV, CDV and CCoV. The coefficient of infection (COI), indicating the concentration of the virus in the sample (positive correlation between COI and viral concentration in the sample), was determined. A complete blood count (CBC) analysis was also performed to determine the numbers of red blood cells (RBC), white blood cells (WBC) and platelets (PLT). The cytological examination of the blood was performed as a complementary laboratory test. Beyond providing valuable information about blood cells, the smear was also examined for the presence of *Mycoplasma* spp., *Anaplasma* spp., and *Babesia* spp., respectively.

### 2.3 Viral RNA extraction and SARS-CoV-2 RT-PCR

Nucleic acids were extracted from the OPS, NS and RS with an NucliSENS EasyMAG instrument (BioMérieux, Lyon, France) and the TaqPath™ COVID-19 RT-PCR assay (Thermo Fisher Scientific, Waltham, Massachusetts, USA) targeting three viral genes (ORF1ab, S and N) was performed according to the manufacturer’s instructions, to detect SARS-CoV-2 RNA. All positive samples were confirmed by repeating the nucleic acid extraction process and RT-PCR testing. Samples for which the second RT-PCR was positive for SARS-CoV-2 were considered positive and those for which the second test was negative were considered inconclusive.

### 2.4 Antibodies against the N protein of SARS-CoV-2

Serum samples were tested for the presence of immunoglobulins (Ig) against the SARS-CoV-2 nucleocapsid (N) with a double-antigen ELISA kit (ID Screen® SARS-CoV-2 Double Antigen Multi-species, Innovative Diagnostics, Grabels, France). In accordance with the manufacturer’s instructions, samples with an inhibition index greater than 60% were considered positive.

### 2.5 Virus-neutralizing test (VNT)

Neutralizing antibody (NAb) titers were assessed only for serum samples with an inhibition index greater than 10% in a whole-virus replication assay with a clinical strain of SARS-CoV-2 (GenBank accession number MW322968) as previously described (21). Briefly, serum samples were decomplemented by heat inactivation (56 °C for 30 min), subjected to three-fold serial dilution (starting at 1:10 to 1:810) in duplicate, and incubated with 50 μL of a viral dilution (2 × 10^3^ TCID_50_/ml) in a 96-well plate at 37 °C for 60 min. We then added 100 μL of a suspension of Vero E6 cells (3 × 10^5^ cells/ml) to the mixture and incubated at 37 °C under an atmosphere containing 5% CO_2_ for 4 days. A microscopy examination was performed to assess the cytopathic effect (CPE) on day 4. An infectivity score was assigned to each well: 0, no cytopathic effect; 1, a fraction of the cells affected; and 2, all of the cells affected. The scores for the two replicates were added and transformed into a percentage of the maximum score (e.g., score of 4 = 100%). The same known positive control serum was added to each experiment to assess repeatability. NAb titers are expressed as the highest serum dilution inhibiting the CPE by 90% (NT_90_). They were inferred by non-linear regression with a four-parameter variable slope model in GraphPad Prism 8.0.2 software. Samples with an NT_90_ above 10 were considered to be neutralizing whereas those with an NT_90_ below 10 but non-zero were considered to be partially neutralizing.

### 2.6 Statistical analysis

Prevalence was expressed as proportions with 95% confidence intervals (95% CI). Categorical variables (species and sex) were compared using Fisher’s exact test, and odds ratios (OR) with 95% CI were calculated. Age distribution between the VNT-positive and VNT-negative animals was compared using the Mann–Whitney U test. A *p*-value <0.05 was considered statistically significant. Data were analyzed using GraphPad Prism 10 (version 10.5.0, GraphPad Software, Boston, Massachusetts, USA).

## 3 Results

From April 2021 to July 2022, 99 dogs and 41 cats belonging to owners with prior or ongoing SARS-CoV-2 infections presenting at the Iasi Faculty of Veterinary Medicine were included.

Among these 140 pets enrolled in the study, 45 (32.14%) were vaccinated, of which 14 (10%) were cats and 31 (22.14%) were dogs. However, most of them (90/140, 64.29%) were not vaccinated, comprising 26 (18.57%) cats and 64 (45.71%) dogs. A small proportion of pets (5/140, 3.57%) underwent partial or incomplete immunization, consisting of one cat (0.71%) and four (2.86%) dogs.

Concerning the lifestyle of the pets, 37 out of 140 (26.43%) were living exclusively indoors, including 19 (13.57%) cats and 18 (12.86%) dogs. Most of the pets (65/140, 46.43%) were housed exclusively outdoors, of which 11 (7.86%) were cats and 54 (38.57%) were dogs. Pets with a mixed lifestyle accounted for 27.14% (38/140), comprising 11 (7.86%) cats and 27 (19.28%) dogs.

According to the questionnaire completed by the pet owners, no travel history in the past year was reported for cats (0%). Regarding dogs, a travel history to Europe was mentioned in only one (0.71%) out of 99 cases.

Regarding other pets in the household, 47 out of 140 (33.57%) owners, all of them dog owners, reported the presence of another dog. In addition, 22 out of 140 (15.71%) owners, including 18 (12.86%) cat owners and four (2.85%) dog owners, reported the presence of another cat. Twenty out of 140 (14.29%) pet owners, including 13 (9.28%) cat owners and 7 (5%) dog owners, confirmed the presence of both cats and dogs in the household. In contrast, 51 out of 140 (36.43%) owners reported not sharing their household with other pets.

Plasma, oropharyngeal, nasal and rectal samples were collected for SARS-CoV-2 serological and RT-PCR assays. For the 140 companion animals tested by RT-PCR on rectal (*n* = 139), oropharyngeal (*n* = 136) and nasal (*n* = 122) swabs, only one cat (0.7%; 95% CI: 0.1–4) tested positive for SARS-CoV-2. The positive result was obtained from an oropharyngeal swab, 10 days after the onset of COVID-19 in the owner, and viral load was low (specimen #1, cycle threshold at 36). The cat was clinically healthy and was in direct contact with the SARS-CoV-2-positive owner, who presented respiratory symptoms.

Antibodies against SARS-CoV-2 nucleocapsid (anti-N antibodies) were detected in 12 of 137 (8.8%; 95% CI: 5.2–14.9) companion animals, with a prevalence of 12.5% (95% CI: 5.5–26.1) and 7.2% (95% CI: 3.6–14.4) for cats and dogs, respectively (Table 1). Five of the seropositive pets were clinically healthy (3 cats and 2 dogs). Three of the seropositive animals were dogs diagnosed with CPV (*n* = 2) or CDV (*n* = 1) infections. The remaining four animals testing positive for anti-N antibodies were a cat diagnosed with lung adenocarcinoma, a cat with pulmonary strongyloidiasis, a dog with hemopericardium, hydropericardium, splenic and bladder tumors and a dog with an infiltrative myocardial disease (Suppl. Table). All of these animals had close contact with their owners during ongoing or prior SARS-CoV-2 infections. The two CPV-positive dogs (specimens #8 and #9) were admitted to the clinic with similar clinical signs (vomiting, bloody diarrhea, lethargy and loss of appetite) typical of CPV infection. One of these dogs was also found to have a concomitant *Mycoplasma haemocanis* infection. Both dogs recovered fully on supportive treatment. On presentation, the dog diagnosed with CDV infection (specimen #9) had ataxia, a lack of coordination and loss of appetite, symptoms specific to this nervous condition. Given the lifelong neurological symptoms, the dog was euthanized at the owner’s request. The other companion animals presenting symptoms had a poor appetite, somnolence, hoarseness, dyspnea and abdominal breathing (specimen #3), with apathy, vomiting and heart disorders (specimen #4), with a very poor body condition leading to a request for euthanasia (specimen #6) or with chronic dry cough and asphyxia, of about 6 months’ duration (specimen #7). Dog #4 was diagnosed with infiltrative myocarditis.

**Table 1:** Prevalence of SARS-CoV-2 RNA, anti-nucleocapsid antibodies and neutralizing antibodies in dogs and cats from Romania.

Characteristics	Total, no. (%)	Dog, no. (%)	Cat, no. (%)
Demography	140 (100)	99 (70.7)	41 (29.3)
Sex (M)	77 (55)	52 (52.5)	16 (39)
Age, mean (year) [IQR]	1.8 [0.5–5.8]	1.6 [0.5–6.0]	1.8 [1.0–4.0]
SARS-CoV-2 RT-PCR			
Negative	139 (99.3)	99 (100)	40 (97.6)
Positive	1 (0.7)	0 (0)	1 (2.4)
Anti-N antibodies			
Negative	125 (91.2)*	90 (92.8)*	35 (87.5)*
Positive	12 (8.8)*	7 (7.2)*	5 (12.5)*
VNT†	32 (100)	22 (68.8)	10 (31.3)
NAbs Negative	20 (62.5)	16 (72.7)	4 (40)
NAbs Positive	12 (37.5)	6 (27.3)	6 (60)
GMNT_90_ [IQR]	22.9 [8.9–34.1]	11.6 [8.8–14.5]	45.1 [26.4–113.9]

GMNT, geometric mean neutralizing titer 90%; IQR, interquartile range; NAbs, Neutralizing antibodies; RT-PCR, reverse transcription polymerase chain reaction; VNT, virus neutralization test; *, Serum samples were not available for two dogs and one cat; †, VNT were only performed on sera displaying an anti-N antibodies inhibition index greater than 10%.

Samples with a SARS-CoV-2 anti-N inhibition index above 10% (*n* = 32) were subjected to a VNT. All five anti-N-seropositive cats gave positive results in the VNT, whereas the presence of neutralizing antibodies was confirmed for only three of the seven anti-N-seropositive dogs (specimens #4, #9 and #10). Moreover, another three dogs and one cat were found to have SARS-CoV-2 NAbs despite being below the anti-N inhibition index threshold set by the manufacturer (Table 1). These companion animals were mostly healthy at the time of the study (Supplementary Table). Overall, 12 animals (8.6%; 95% CI: 5.0–14.4%) tested positive by VNT. The proportion of cats harboring NAbs was 14.6% (6/41), compared with 6.1% (6/99) in dogs. However, this difference did not reach statistical significance (OR = 1.825, 95% CI: 0.544–6.125, *p* = 0.334). No significant association was found between sex and VNT positivity but age was significantly associated with VNT status: an increasing age was linked to a lower likelihood of being VNT positive (OR = 0.685 per year, 95% CI: 0.475–0.989, *p* = 0.043) (Table 2). Interestingly, the geometric mean NT_90_ (GMNT_90_) [interquartile (IQR)] was higher in cats (45.1 [26.4–113.9]) than in dogs (GMNT_90_ of 11.6 [8.8–14.5]) (Table 1).

**Table 2:** Univariate analyses for factors associates with the neutralizing antibodies positivity.

Variables	Univariate analysis		
	Odd ratio	CI 95%	*p*-value
Species (control = cat)	1.825	0.544–6.125	0.334
Sex (control = male)	0.803	0.246–2.623	0.768
Age	0.685	0.475–0.989	0.043

One healthy cat (specimen #2) with follow-up serum samples collected between 8 and 15 days after the onset of COVID-19 in its owner, underwent seroconversion for anti-N antibodies, with the emergence of NAbs despite all the samples from this cat remaining RT-PCR-negative for SARS-CoV-2 (Supplementary Table). The other five cats from the same household remained seronegative for anti-N antibodies and none had positive RT-PCR results for their follow-up samples.

## 4 Discussion

In this study, we investigated SARS-CoV-2 exposure in companion animals from households with confirmed human COVID-19 cases. Viral RNA was detected in only one cat, and NAbs were identified in both cats and dogs. The overall VNT seroprevalence was 8.8%, with a higher proportion observed in cats (12.5%) compared with dogs (7.2%). Although this difference did not reach statistical significance, it is consistent with previous evidence that cats are more susceptible to SARS-CoV-2 infection than dogs. Worldwide, several seroprevalence studies have been conducted in pets, showing variable results. In cats, antibodies against SARS-CoV-2 have been identified at prevalences ranging from 0 to 21.7% (10, 13, 15, 22–26), whereas lower estimates have been reported in dogs, ranging from 0 to 14.5% (11, 13, 15, 22, 26–32). In eastern Europe, few data are available on pet’s serological studies. In Serbia, a neighboring country, 69 dogs and 36 cats were tested for anti-SARS-CoV-2 Ab between 2020 and 2021 revealing a lower seropositivity of 1.45% in dogs and 5.56% in cats (33). Similarly, a larger study from Croatia comprising 131 cats and 656 dogs demonstrated a low seropositivity of only 0.76% in cats and 0.31% in dogs (31).

The presence of Nabs in animals without detectable viral RNA is most likely the result of prior exposure, as viral shedding in pets is short-lived, generally less than 1 week (34). NAbs detection therefore remains a valuable tool for identifying previous infection. However, the clinical significance of neutralizing antibodies in the absence of viral RNA detection is not yet clear. While such antibodies may indicate some degree of protection, they do not provide direct information on the risk of onward transmission. One cat displayed NAbs despite being seronegative for anti-N antibodies. This particular profile may be explained by a loss of anti-N antibodies, as observed in humans. Indeed, despite testing negative for anti-N antibodies according to the threshold set by the manufacturer, this cat had an anti-N inhibition index greater than those of the other anti-N antibody-negative pets, suggesting that this feature could be used as a proxy for the decline of anti-N antibodies.

Another interesting point was the possible link between prior SARS-CoV-2 infection and a myocardial condition; myocarditis associated with multisystem inflammatory syndrome is a well-recognized complication of COVID-19 in humans and cats (35). One of the seropositive dogs (specimen #4) was diagnosed with infiltrative myocardial disease about 1 month after the onset of COVID-19 in its owner. This dog had both anti-N antibodies and NAbs against SARS-CoV-2. Although this remains speculative, we cannot exclude or confirm the possibility that the condition of this dog was related to prior its infection.

We found that NAb titers were higher in cats than in dogs. This finding is consistent with published reports that cats are more susceptible to infection with SARS-CoV-2 than dogs. These data are consistent with those of other studies demonstrating that cats experimentally infected with SARS-CoV-2 can readily transmit the infection to naïve cats, whereas naïve dogs closely confined with experimentally infected dogs do not become infected (36, 37). We demonstrated SARS-CoV-2 seroconversion in one cat (specimen #2) based on tests on three samples collected 8 to 15 days after the onset of COVID-19 in its owner. This case may represent an instance of anthropogenic transmission of SARS-CoV-2 in a cat and suggests that there is a short time window for SARS-CoV-2 replication in cats, as the oropharyngeal, nasal and rectal swabs collected at the same time were all RT-PCR-negative. Nevertheless, the transmission potential of this possible reverse zoonosis remains low, as the other five cats from the same household tested negative in both molecular and serological assays.

Our results are consistent with those reported globally, confirming the ability of SARS-CoV-2 to cross the species barrier, even during the different phases of the pandemic (9, 11, 16). All the pets enrolled in the study resided in the same household as their SARS-CoV-2 positive owner, with whom they maintained close contact. Except for the daily walks (applicable to dogs) for physiological needs, the pets had no contact with other animals or people outside their household members. Except animals diagnosed with different infectious diseases, beyond the disease-specific symptoms, no abnormal behavior was noticed by the owner or veterinarian practitioners.

This study also has limitations. The sample size was modest, particularly for cats, resulting in a limited statistical power to detect associations. The recruitment at the veterinary clinics may have introduced selection bias (convenience sampling) and diagnostic assays also have inherent constrains: PCR positivity is restricted to a narrow window of viral shedding, while serological assays based on anti-N detection may be affected by cross-reactivity, particularly in dogs infected with other coronaviruses, or by a decline of anti-N Ab levels. Our study was conducted during the pre-Omicron and early Omicron circulation period. It has been suggested that early SARS-CoV-2 variants differed in their transmissibility to companion animals compared with Omicron lineages, now dominant worldwide. This temporal context should be considered when interpreting our findings, as circulating variants likely influenced infection dynamics in pets.

In conclusion, this study provides evidence for the anthropogenic transmission of SARS-CoV-2 to pets in Romania, as demonstrated by the molecular confirmation of infection in a household cat, seroconversion in another, and the detection of NAbs in other cats and dogs from owners with COVID-19. These findings highlight the susceptibility of companion animals to SARS-CoV-2 with stronger support for infection in cats during the pre-Omicron and early Omicron era. These results are consistent with global data confirming that companion animals could be infected by SARS-CoV-2 but appeared to have played only a minor role in transmission. Larger, longitudinal studies that integrate variant-specific data are necessary to better understand the implication of such transmission.

## Data Availability

The original contributions presented in the study are included in the article/Supplementary material, further inquiries can be directed to the corresponding author.
