# Supplementary material for: SARS-CoV-2 infection and exposure in cats and dogs in Romania
Source: Front Vet Sci. 2025 Oct 29;12:1671681. doi: 10.3389/fvets.2025.1671681 (PMC12605256; doi:10.3389/fvets.2025.1671681)
Supplement: Supplementary file 1 [file Table_1.docx]

**Supplemental Table.** Clinical and biological characteristics of SARS-CoV-2 infected or seropositive pets

| ID | Species | Diagnosis | Time between owner’s onset and animal sampling | Clinical pathology results | SARS-CoV-2 RT-PCR | Anti-N  Abs  result | Anti-N Abs index | Nabs | NT90 |
| --- | --- | --- | --- | --- | --- | --- | --- | --- | --- |
| 1 | Cat | None (healthy) | 10 days | Monocytosis | Positive (OPS) | Negative | -10.24 | N/A | N/A |
| 2 | Cat | None (healthy) | 8 days | Neutrophilia, lymphopenia, monocytosis, thrombocytopenia. Mycoplasma and Anaplasma negative. | Negative | Negative | 23.14 | No | 0 |
| 2 | Cat | None (healthy) | 12 days | Neutrophilia, lymphopenia, monocytosis, thrombocytopenia. Mycoplasma and Anaplasma negative. | Negative | Negative | 47.69 | No | 0 |
| 2 | Cat | None (healthy) | 15 days | Neutrophilia, lymphopenia, monocytosis, thrombocytopenia. Mycoplasma and Anaplasma negative. | Negative | Positive | 62.81 | Partially | 8.4 |
| 3 | Cat | Lung adenocarcinoma | 1.5 month | Neutrophilia, lymphopenia, eosinopenia. Mycoplasma and Anaplasma negative. | Negative | Positive | 470.11 | Yes | 136.31 |
| 4 | Dog | Infiltrative myocardial pathology | 1 month | Neutrophilia, high red blood cells count. | Negative | Positive | 717.04 | Yes | 15.92 |
| 5 | Cat | None (healthy) | 1 month | Neutropenia, lymphocytosis, eosinophilia. | Negative | Positive | 99.47 | Yes | 26.44 |
| 6 | Dog | Haemopericardium, hydropericardium, splenic and bladder tumors | 4 months | Neutrophilia, lymphopenia, thrombocytopenia, anemia. Mycoplasma and Anaplasma negative. | Negative | Positive | 146.60 | No | 0 |
| 7 | Cat | Pulmonary strongyloidosis | 2 months | Neutropenia, lymphocytosis. | Negative | Positive | 67.03 | Yes | 26.44 |
| 8 | Dog | CPV | 4 months | Monocytosis | Negative | Positive | 407.21 | No | 0 |
| 9 | Dog | Carre disease | 4 months | Anemia, thrombocytopenia. | Negative | Positive | 95.89 | Partially | 6.67 |
| 10 | Dog | None (healthy) | 6 months | CPV and CCoV negative. | Negative | Positive | 802.00 | Yes | 29.9 |
| 11 | Dog | CPV | 6 months | Neutropenia, lymphopenia, eosinopenia, monocytosis, anisocytosis. *Mycoplasma haemocanis* positive. | Negative | Positive | 168.25 | No | 0 |
| 12 | Cat | None (healthy) | 3 months | N/A | Negative | Positive | 794.98 | Yes | 226.32 |
| 13 | Dog | None (healthy) | 2 months | N/A | Negative | Positive | 731.19 | No | 0 |
| 14 | Dog | Stroke suspicion | 10 days | Neutrophilia, lymphopenia, monocytosis. | Negative | Negative | 33.02 | Partially | 8.96 |
| 15 | Cat | None (healthy) | 3 months | N/A | Negative | Negative | 21.97 | Yes | 46.59 |
| 16 | Dog | None (healthy) | 2 months | N/A | Negative | Negative | 33.10 | Partially | 8.70 |
| 17 | Dog | None (healthy) | 1.5 months | Monocytosis | Negative | Negative | 53.85 | Yes | 10.08 |

Abs: Antibodies; NAbs: neutralizing antibodies; NT90: neutralizing titer at 90%; OPS: oropharyngeal swab; CPV: canine parvovirus; CCoV: canine coronavirus.
